# Impact of COVID-19 on elective, emergency and oncological surgery during the first and the second wave in a tertiary university hospital: Have we learned the lessons?

**DOI:** 10.1007/s00508-022-02041-y

**Published:** 2022-05-24

**Authors:** Lukas Gasteiger, Julia Abram, Sebastian Klein, Pia Tscholl, Tobias Hell, Gabriel Putzer, Berthold Moser, Michael Joannidis, Judith Martini

**Affiliations:** 1 Department of Anaesthesiology and Intensive Care Medicine, Medical University of Innsbruck, Innsbruck, Austria; 2 Division of Intensive Care and Emergency Medicine, Department of Internal Medicine, Medical University of Innsbruck, Innsbruck, Austria; 3 Department of Mathematics, Faculty of Mathematics, Computer Science and Physics, University of Innsbruck, Innsbruck, Austria; 4 Department of Anaesthesia, See-Spital Horgen, Horgen, Switzerland

**Keywords:** Acute surgery, Elective surgery, Public health, Collateral damage syndrome, COVID-19

## Abstract

**Background:**

The COVID-19 pandemic caused an important reduction in surgical activities during the first wave. Aim of this retrospective time-trend analysis was to examine whether also during the second wave in fall and winter 2020/2021 surgical interventions decreased.

**Methods:**

Absolut numbers and types of surgeries in a tertiary university hospital during the second COVID-19 wave in fall/winter 2020/2021 were collected from the surgical planning software and compared with the same time frame over the last 5 years. In a second step, the reduction of surgical interventions during the second wave was compared with the reduction of surgical procedures during the first wave in spring 2020 at the same hospital.

**Results:**

Despite a higher 7‑day incidence of COVID-19 infection and a higher number of patients needing ICU treatment during the second wave, the reduction of surgical interventions was 3.22% compared to 65.29% during the first wave (*p* < 0.0001). Elective surgical interventions decreased by 88.63% during the first wave compared to 1.79% during the second wave (*p* < 0.0001). Emergency and oncological interventions decreased by 35.17% during the first wave compared to 5.15% during the second wave (*p* : 0.0007) and 47.59% compared to 3.89% (*p* < 0.0001), respectively. Surgical activity reduction in our institution was less pronounced despite higher occupancy of ICU beds during the second COVID-19 wave in fall/winter 2020/2021.

**Conclusion:**

Better understanding of the disease, adequate supply of disposables and improved interdisciplinary day by day management of surgical and ICU resources may have contributed to this improvement.

## Introduction

The COVID-19 pandemic has brought healthcare systems around the world face to face with unknown challenges. Also, Austria with 8 million inhabitants was severely affected by the pandemic. By April 2022 more than 3.9 million individuals had tested positive for SARS-CoV‑2 and to date more than 15,000 deaths can be directly attributed to the infection [1].

One side effect of the so-called first wave of the pandemic was that frequencies of elective and oncologic surgeries were severely influenced, as during this period a drastic reduction in elective surgery was advised [2]. Anesthesiology and critical care departments often share medical personnel; in order to provide sufficient resources, staff members from the operating theaters were shifted to intensive care units [3, 4]. Even if efforts were undertaken to prevent insufficient supply for other patients needing medical treatment, countless studies have described a massive reduction in emergency and cancer surgery [2, 5, 6]. To date, the impact of the pandemic on public health is not completely clear but many authors agree that due to interventions such as the suspension of cancer screening programs and the prioritization only for urgent symptomatic cases, as was the practice in some countries in spring 2020, a substantial increase in potentially avoidable damage and also cancer deaths have to be expected [7, 8].

Innsbruck Medical University Hospital, Austria, is a tertiary hospital in Tyrol (750,000 inhabitants). Including surrounding areas, it provides the highest level of care for approximately 1.8 million people and is also centrally involved in the regional COVID-19 network to cover ICU demand during the pandemic [9]. During the first lockdown in spring 2020 a drastic reduction in elective and oncological surgical activities was observed as compared to the same period during the previous 5 years [2]. As some characteristics of the two first waves of the COVID-19 pandemic strongly differed, such as different measures of social restriction, general knowledge on the nature of the disease, availability of disposables and medical supply, a difference in the overall amount of surgical activity should be expected. On the other side the number of patients needing ICU-treatment and the 7‑day incidence in Tyrol was more accentuated during the second wave compared to the first one. Therefore, aim of this retrospective time-trend analysis was to assess if during the second COVID-19 pandemic wave in fall/winter 2020/2021 a comparable reduction rate in elective, emergency and oncologic surgeries occurred as during the first wave in spring 2020 [10].

## Material and methods

### Data collection and statistical analysis

Ethics approval (EK Nr: 1124/2020, dated 2020_05_17, and EK Nr: 1099/2020, dated 2020-04-28) for this retrospective assessment was obtained from the local ethics committee (*Ethikkommission der Medizinischen Universität Innsbruck*—Austria). The methods and the study design were assumed from our previous study [2].

Again, data on all surgical activities during the two pandemic waves were collected from the surgical planning software myMedis (Getinge, IT Solutions GmbH, Getinge, Sweden). These data were compared with the mean numbers for the corresponding periods during the previous 5 years. The first collecting period was 15 March–14 April 2020 (lockdown 1). The corresponding period was 15 March–14 April 2015–2019 (corresponding period 1). The second sampling period was 12 October 2020–15 January 2021 (peak period of the second wave in Tyrol). The corresponding period was 12 October–15 January during the years 2015/2016 to 2019/2020 (corresponding period 2). The second sampling period was defined according to the necessity to readapt daily evaluations for surgical interventions at our institution because of increased 7‑day incidence of newly detected SARS-CoV‑2 infections.

The primary outcome of this study was to assess the rate of reduction of surgical procedures during the first wave (15 March–14 April 2020) and second wave (12 October 2020–15 January 2021) of the COVID-19 pandemic at our institution.

For every patient baseline characteristics, such as gender, age, country of origin and comorbidities were recorded. Comorbidities and overall physical health were rated using the American Society of Anesthesiologists Physical Status classification system (ASA status). In agreement with our previously published trial, all interventions were classified according to the organizational unit, date and time of surgery (core time: 07:00–17:00, shift time: 17:00–07:00). The subdivision of all interventions into elective, emergency or oncologic interventions was conducted by two medical students under the supervision of two consultant anesthesiologists. If there was no unanimous agreement, a second round of independent review by other two consultant anesthesiologists was carried out.

Emergency surgeries were defined as surgical procedures that need to be performed within 24 h (e.g., aortic dissection, mechanical ileus).

The results describing the amount and type of reduction of surgical activities in the first lockdown period have already been published [2]. To evaluate the management of the various phases and the dynamics of the pandemic, we describe these previous data again and compare the changes in the two waves in a second step.

Specifically, we compared the number of surgical activities (overall, emergency, elective, oncologic) of each of the two periods with the mean of the same period over the last 5 years. By doing so we were able define the rate of reduction for the specific wave (first and second) compared to the mean of the same period over the last 5 years. In a second step we compared the reductions of surgical activities between the two waves.

We also integrated the numeric course of SARS-CoV‑2-infected patients in Tyrol and of critically ill SARS-CoV‑2 patients treated at our hospital ICU.

Statistical analyses were conducted using R, version 4.0.5 (The R Foundation for Statistical Computing, c/o Institute for Statistics and Mathematics, Wirtschaftsuniversität Wien, Vienna, Austria). All statistical assessments were two-sided and a significance level of 5% was used. We present the absolute number of surgeries with a 95% confidence interval (CI) for the period 2015–2019 and absolute numbers for 2020/2021. Likewise for the first and second wave, other categorical variables are presented as frequencies (%) and continuous data as mean (95% CI). We applied the Exact Poisson test to assess the difference in the number of surgeries between 2015–2019 and 2020/2021 as well as between the first and second wave, Fisher’s exact test for binary variables and the Welch two sample t‑test for continuous variables. We show effect size as odds ratios (OR) for binary variables and estimated median difference for the continuous variable age, with 95% CIs.

## Results

### Patient demographics, comorbidities, and country of origin

During the second wave the mean age of the patients was significantly higher than during the previous 5 years (56.6 vs. 55 years; *p* < 0.0001). (Table 1).

**Table 1:** Patient demographics, comorbidities, and patient origin during the second wave compared to corresponding period 2015/2016–2019/2020

	Mean 2015/2016–2019/2020 with 95% CI^a^ (*n* = 57,337)	2020/2021 (*n* = 11,082)	Estimate with 95% CI^b^	Decrease (%)^c^	*p* value^d^	Missing
Age (years)	55 (54.9 to 55.2)	56.6 (56.2 to 57.1)	−1.6 (−2.1 to −1.1)	−2.93 (−3.94 to −1.92)	< 0.0001	0/0
Gender (female)	30,159/57,337 (52.6%)	5849/11,082 (52.8%)	1.01 (0.97 to 1.05)	−0.37 (−1.56 to 0.82)	0.7317	0/0
Tyrolean	51,965/57,337 (90.6%)	10,228/11,082 (92.3%)	1.24 (1.15 to 1.34)	−1.85 (−2.54 to −1.15)	< 0.0001	0/0
ASA score= 1	10,838/35,817 (30.3%)	1854/7137 (26%)	0.81 (0.76 to 0.86)	11.48 (6.92 to 16.03)	< 0.0001	3945/21,520
ASA score = 2	14,825/35,817 (41.4%)	3059/7137 (42.9%)	1.06 (1.01 to 1.12)	−8.49 (−25.84 to 8.87)	0.0221	3945/21,520
ASA Score = 3	9271/35,817 (25.9%)	2006/7137 (28.1%)	1.12 (1.06 to 1.19)	−13.35 (−28.68 to 1.98)	0.0001	3945/21,520
ASA Score = 4	822/35,817 (2.3%)	199/7137 (2.8%)	1.22 (1.04 to 1.43)	−27.26 (−47.36 to −7.16)	0.0136	3945/21,520
ASA Score = 5	61/35,817 (0.2%)	19/7137 (0.3%)	1.56 (0.88 to 2.66)	−85.54 (−177.16 to 6.07)	0.097	3945/21,520

^a^ Binary data are presented as no./total no. (%)

^b^ Odds ratios for binary variables and estimated mean difference for continuous variables

^c^ Estimated mean difference with standard parametric CI

^d^ Assessed by Fisher’s exact test

There was a significant reduction in patients scored as ASA 1 (26% vs. 30.3% *p* < 0.0001) and significantly more patients were scored as ASA 2, 3 or 4 (ASA 2: 42.9% vs. 41.4%; *p* = 0.0221; ASA 3: 28.1% vs. 25.9% *p* < 0.0001; ASA 4: 2.8% vs. 2.3%; *p* = 0.0136) compared to the previous 5 years. (Table 1).

Significantly more patients were inhabitants of Tyrol (92.3% vs. 90.6%, respectively; *p* < 0.0001). (Table 1).

### Numbers and timing of surgical interventions

During the second wave a total of 11,082 surgeries were performed compared to a mean of 11,467.4 in the corresponding period, thus giving a reduction of 3.22% (*p* < 0.0001). Also, a significant reduction in elective surgeries from 6579.6 to 6460 (1.79%, *p* < 0.0001) and emergency surgical interventions from 3645.4 to 3432 (5.15%, *p* < 0.0001) was observed. Equally, a reduction in oncological surgeries from 1239.8 to 1186 (3.89%, *p* < 0.0001) was observed during the second wave. (Table 2).

**Table 2:** Overall number of surgeries, numbers for elective, emergency and oncologic surgery and timing (core time, shift time) during the second wave compared to corresponding period 2015/2016–2019/2020

	Mean 2015/2016–2019/2020 with 95% CI^a^	2020/2021	Estimate with 95% CI^b^	Decrease (%)^c^	*p* value^d^
Total number of surgeries	11,467.4 (11,373.7 to 11,561.7)	11,082	385.4 (291.7 to 479.7)	3.22 (−1.89 to 8.32)	< 0.0001
Elective surgeries	6579.6 (6508.7 to 6651.1)	6460	119.6 (48.7 to 191.1)	1.79 (−0.34 to 3.93)	0.0009
Emergency surgeries	3645.4 (3592.7 to 3698.7)	3432	213.4 (160.7 to 266.7)	5.15 (−5.7 to 16)	< 0.0001
Oncological surgeries	1239.8 (1209.1 to 1271.1)	1186	53.8 (23.1 to 85.1)	3.89 (−5.06 to 12.84)	0.0005
Core time surgeries	10,010 (9922.5 to 10,098.1)	9776	234 (146.5 to 322.1)	2.2 (−2.89 to 7.28)	< 0.0001
Shift time surgeries	1457.4 (1424.1 to 1491.3)	1306	151.4 (118.1 to 185.3)	10.12 (3.36 to 16.89)	< 0.0001

^a^ Estimated mean based on a Poisson distribution

^b^ Estimated mean difference based on a Poisson distribution

^c^ Mean decrease calculated 100/(number of surgeries during comparison period) * (number of surgeries during comparison period − number of surgeries during lockdown period) with standard parametric CI for mean

^d^ Assessed by Poisson test

A total of 9776 surgeries were performed during core time compared to 10,010 during the previous 5 years, which results in a reduction of 2.2% (*p* < 0.0001). Shift time interventions were also reduced by 10.12% (*p* < 0.0001) from 1457.4 to 1306 (Table 2).

### Oncological surgery

In total 1186 oncological surgeries were performed during the observed time (second wave). The mean number during the previous 5 years was 1239.8, resulting in a reduction of 3.89% (*p* = 0.0005).

**Fig. 1:**
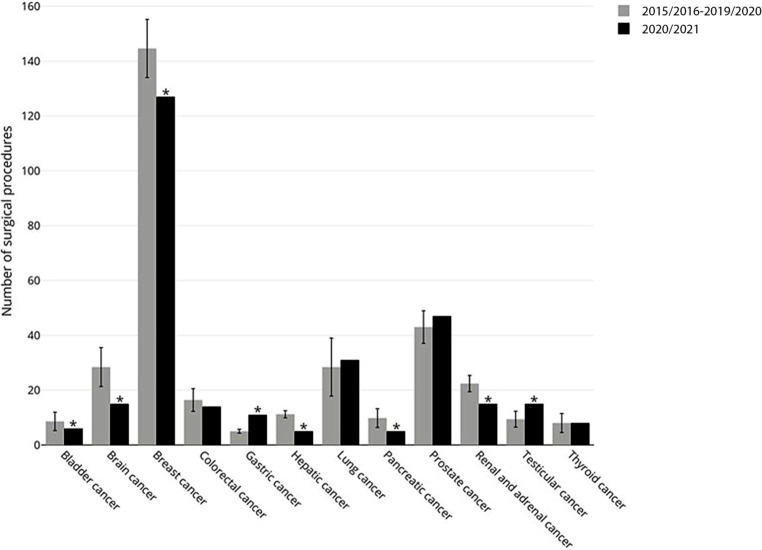
Detailed analyses for specific oncological surgeries

When oncological surgery was analyzed for single entities, divergent results were observed. A significant reduction was found for breast, hepatic, pancreatic, (supra) renal, bladder, retroperitoneal cancer and tumor craniectomies. In contrast, testicular and gastric cancers were surgically treated more often; some tumor entities remained unchanged. (Fig. 1).

### Comparison of the first and the second wave

As the two periods were of different length (4 weeks versus 11 weeks) we mostly provide percentage and not absolute numbers for the comparison of the two periods. A comparison of the baseline characteristics of patients treated during the first and second COVID-19 pandemic waves showed no difference in gender. Patients were significantly older during the second wave as compared to the first wave (55.6 vs. 54.8 years, respectively; *p* < 0.0001) and the percentage of patients originating from Tyrol decreased during the second wave (92.3% vs. 94.2% respectively; *p* = 0.0081). (Table 3).

**Table 3:** Comparison of the first and second wave: baseline characteristics of the patients and patient origin (Tyrolean inhabitants)

	First wave (*n* = 1391)^a^	Second wave (*n* = 11,082)	Estimate with 95% CI^b^	*p* value^c^	Missing
Age (years)	54.8 (53.7 to 55.9)	56.6 (56.2 to 57.1)	−1.9 (−3 to −0.7)	0.0019	0/0
Gender (female)	755/1391 (54.3%)	5849/11,082 (52.8%)	0.94 (0.84 to 1.05)	0.2919	0/0
Tyrolean	1311/1391 (94.2%)	10,228/11,082 (92.3%)	0.73 (0.57 to 0.93)	0.0081	0/0
ASA score = 1	227/1046 (21.7%)	1854/7137 (26%)	1.27 (1.08 to 1.49)	0.003	345/3945
ASA score = 2	397/1046 (38%)	3059/7137 (42.9%)	1.23 (1.07 to 1.41)	0.0028	345/3945
ASA score = 3	360/1046 (34.4%)	2006/7137 (28.1%)	0.75 (0.65 to 0.86)	< 0.0001	345/3945
ASA score = 4	55/1046 (5.3%)	199/7137 (2.8%)	0.52 (0.38 to 0.72)	0.0001	345/3945
ASA score = 5	6/1046 (0.6%)	19/7137 (0.3%)	0.46 (0.18 to 1.42)	0.1228	345/3945

^a^ Binary data are presented as no./total no. (%)

^b^ Odds ratios for binary variables and estimated mean difference for continuous variables

^c^ Assessed by Fisher’s exact test

The overall number of interventions decreased by 65.29% during the first wave compared to 3.22% during the second wave (*p* < 0.0001). (Table 4).

**Table 4:** Comparison of the first and second wave: reduction rate overall and for elective, emergency and oncologic surgery. Timing of surgery (shift time, core time)

	Mean decrease total^a^ with 95% CI	Mean decrease first wave with 95% CI	Mean decrease second wave with 95% CI	Estimate with 95% CI^b^	P value^c^
Total number of surgeries	34.25 (10.75 to 57.76)	65.29 (62.73 to 67.85)	3.22 (−1.89 to 8.32)	62.1 (57 to 67.1)	< 0.0001
Elective surgeries	45.21 (12.46 to 77.96)	88.63 (87.6 to 89.65)	1.79 (−0.34 to 3.93)	86.8 (84.7 to 88.9)	< 0.0001
Acute surgeries	20.16 (8 to 32.32)	35.17 (31.14 to 39.2)	5.15 (−5.7 to 16)	30 (19.4 to 40.7)	0.0007
Oncological surgeries	25.74 (8.82 to 42.66)	47.59 (43 to 52.18)	3.89 (−5.06 to 12.84)	43.7 (34.8 to 52.6)	< 0.0001
Core time surgeries	35.56 (10.31 to 60.82)	68.93 (66.38 to 71.48)	2.2 (−2.89 to 7.28)	66.7 (61.7 to 71.8)	< 0.0001
Shift time surgeries	23.24 (12.93 to 33.54)	36.35 (33 to 39.71)	10.12 (3.36 to 16.89)	26.2 (19.5 to 32.9)	0.0001

^a^ Compared to the corresponding time period from 2015 onwards as 100/(number of surgeries during comparison period) * (number of surgeries during comparison period − number of surgeries during COVID period) with standard parametric CI for mean

^b^ Estimated mean difference with standard parametric CI

^c^ Assessed by Welch two sample t‑test

Elective surgical activities decreased by 88.63% during the first wave compared to 1.79% during the second wave (*p* < 0.0001). For emergency interventions, the decrease was 35.17% during the first wave compared to 5.15% during the second wave (*p* : 0.0007) and for oncologic interventions it was 47.59% compared to 3.89% (*p* < 0.0001), respectively. (Table 4).

The reduction in surgical activities was more pronounced during core time (68.93% during the first wave vs 2.2% during the second wave; *p* < 0.0001) but was also given during shift time (36.35% during the first wave vs 10.12% during the second wave; *p* < 0.0001). (Table 4).

**Fig. 2:**
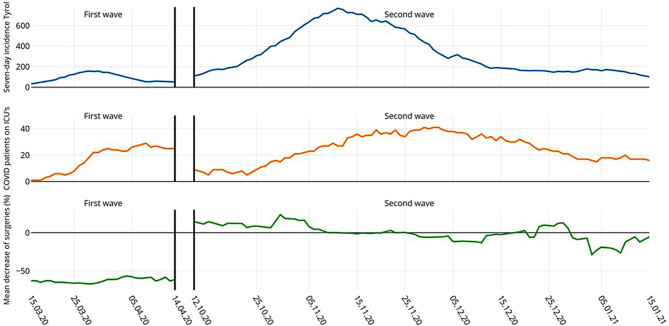
Week by week chronology for both waves in terms of reduction in surgical interventions (mean decrease), 7‑day incidence of SARS-CoV‑2 infections in Tyrol, and number of SARS-CoV‑2 patients treated at our hospital ICUs

During the 4 weeks of the first period of observation 42 COVID-19 patients needed ICU care, whereas during the 11 weeks of the second observation period 178 COVID-19 patients were admitted to one of our hospital’s ICUs. The dynamics for both waves regarding surgical activities, incidence of SARS-CoV‑2 infections in Tyrol and numbers of SARS-CoV‑2 ICU patients treated at our hospital are shown in Fig. 2.

## Discussion

Main result of this retrospective analysis is that in a tertiary university hospital in Austria significantly less surgical interventions were cancelled or postponed during the second COVID-19 pandemic wave compared to the first wave in spring 2020 even though significantly more infected patients were treated on the ICU.

Countless articles have described the severe impact of the early phase of the pandemic on surgical activities and cancer screening programs, when in spring 2020 the first wave of COVID-19 rolled through Europe [8, 11–13]. In an earlier publication we assessed the impact of the first lockdown on the decrease in surgical interventions at our institution and we showed a reduction of 88% in elective surgery and of 35% in emergency surgery [14]. We also reported an overall decline of 47.8% for oncologic surgery. This so called collateral damage syndrome is most likely attributed to the fact that cancer screening measures and cancer diagnosis showed a significant reduction during the first wave of the pandemic in spring 2020 [15].

When comparing the two waves, it becomes evident that we have learned from our earlier experience. In fact, in Tyrol as well as in the rest of Europe the second wave of the COVID-19 pandemic was more pronounced than the first one. The peak 7‑day incidence in Tyrol during the second wave was as high as almost 800 and was reached on 12 November 2020, compared to the first peak of 158 reached on 27 March 2020 [1]. Accordingly, the number of COVID-19-related critically ill patients at our hospital was significantly higher during the second wave in autumn/winter 2020/2021. Nevertheless, the reduction in total surgical activities was only 3.22% compared to the corresponding time during the previous 5 years, whereas surgical activities during the first lockdown in spring 2020 were reduced by 65.4% [2]. This may indicate that a deeper understanding of the disease and better supply with test kits and protective gear led to improved management during the second infection wave and second lockdown; in fact, the reduction of 3.22% lies close to the normal range of variability for surgical activity in our hospital over the last 5 years. This shows that during the second infection wave surgical activities almost returned to average pre-COVID-19 numbers. It also may be worthwhile to report that with the rise of the second wave a broad interdisciplinary day by day crisis management was implemented in our hospital. This interdisciplinary council composed of members of the hospital management, surgeons, anesthesiologists, intensive care physicians and infectiologists met daily from the moment on when more than 8 patients where admitted to the ICU because of COVID-19 (corresponding to 10% of adult ICU capacity). This council was authorized to decide on the number of surgical procedures that could safely be performed the next day without jeopardizing ICU capacities. These decisions were based on regional forecasts for the development of the pandemic based on mathematical models and on urgency of the planned surgical interventions. In addition, as already happened during the first wave, a regional ICU network was established with the aim to provide a uniform use of capacity of all regional ICUs with the idea to prevent overburdening of single centers [9]. All these measures together including rigorous testing and strictly enforced hygiene concepts gave us the possibility to apply a more flexible day by day decision on the number of surgical procedures which could safely be performed, based on short-term forecasts and actual COVID-19 and non-COVID-19 related ICU demand. This flexible and multidisciplinary approach may in part explain why during the second infection wave numbers of surgical activities were close to the normal range of variability of our hospital. As a result, only a marginal reduction in elective and cancer surgical procedures compared to the first wave and to reports from other European countries was found [4, 16].

In fact, our findings stand in contrast to the results of a nationwide survey in the UK, where a reduction of 50% in surgical activity was described during the second pandemic wave in winter 2020/2021 [4]. Also, a recent study from England and Wales showed an overall reduction of 35% in surgical interventions for the entire year 2020 [16]. It must be remembered that the time frame analyzed in our first study [2] strictly covered the lockdown period in Tyrol from 15 March–14 April 2020. The data presented in this study, however, cover a significantly longer time frame, namely from 12 October 2020–15 January 2021, which corresponds to the peak of the second wave in Tyrol. A complete lockdown on social life, including closure of gastronomy and shops other than grocery stores and pharmacies, was not put into force for the entire observation period but only for certain weeks. The strict stay at home policy enforced during the first wave in spring 2020 was no longer followed as strictly, which may explain the increased or near-normal number of surgical interventions. In addition, it should be noted that in contrast to the early phase of the pandemic, there was no longer a shortage of medical disposables, and the development of rapid antigen test kits, which were commercially available by this time, led to rigorous testing of all surgical patients and healthcare workers.

The findings that oncological surgeries decreased only by 3.89% compared to the corresponding period in 2015/2016–2019/2020 is interesting. In fact, as discussed for the total number of surgeries, this could have been also caused by normal variability and may not only have been influenced by the COVID-19 pandemic, as some entities of cancer surgeries even increased (gastric cancer, lung cancer, prostate and testicular cancer). On the other hand, this finding may indicate that the provision of basic healthcare services and the participation in cancer screening programs during the second wave almost returned to prepandemic levels.

In terms of reduction of emergency surgeries, only small differences were found between the first lockdown period in spring 2020 and the second wave in fall/winter 2020/2021. A possible explanation could be that also during the second wave tourism from regions outside Austria was forbidden and therefore a smaller number of people pursued accident-prone outdoor activities such as skiing or snowboarding. In addition, a reduction in tourists goes along with a reduction in the number of potential surgical patients as reflected in the 1.89% reduction in patients originating from areas other than Tyrol.

Our study has several limitations. First, given its single center nature results may not be generalizable to other hospitals or other healthcare systems. This fact must be kept in mind when putting our findings in a greater context and may preclude a direct comparison with other healthcare facilities. Second, the retrospective selection of type of surgery even if performed with strict criteria, may represent a bias.

## Conclusion

An improved understanding of the SARS-CoV‑2 infection and a more flexible interdisciplinary management of resources, especially ICU capacities, led to significantly improved management of the second COVID-19 pandemic wave in Tyrol in fall/winter 2020/2021. This resulted in an only marginal reduction in elective and cancer surgical procedures. The knowledge gained during the fall/winter season 2020/2021 may help with the management of forecasted future waves of the COVID-19 pandemic regarding minimizing the COVID-19 collateral damage syndrome.
